# Supplementary figures and images for: Genetic diversity analysis and molecular characteristics of wild centipedegrass using sequence-related amplified polymorphism (SRAP) markers
Source: PeerJ. 2023 Aug 24;11:e15900. doi: 10.7717/peerj.15900 (PMC10460567; doi:10.7717/peerj.15900)

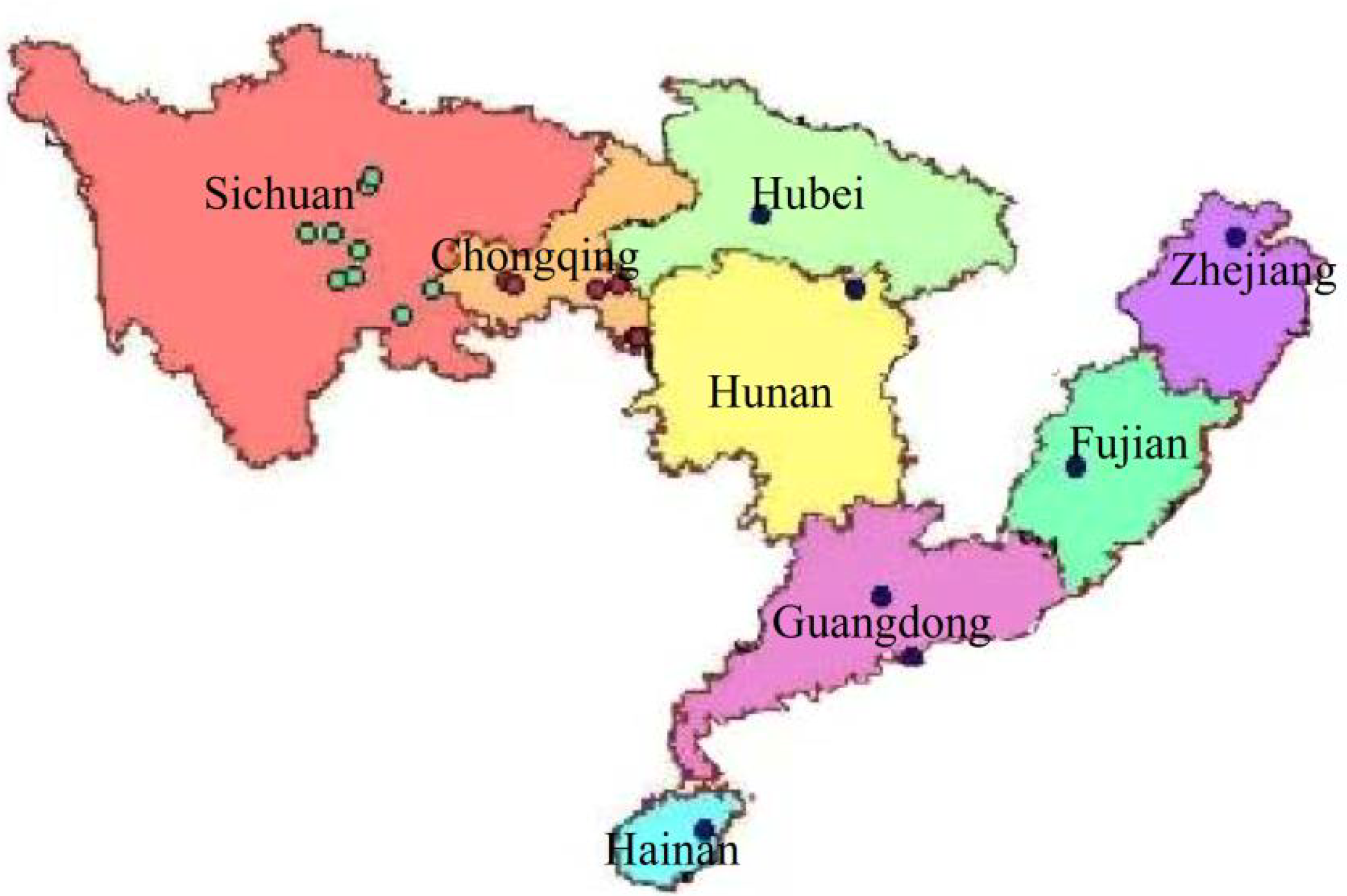

Supplement: Figure S1 [file peerj-11-15900-s001.png]

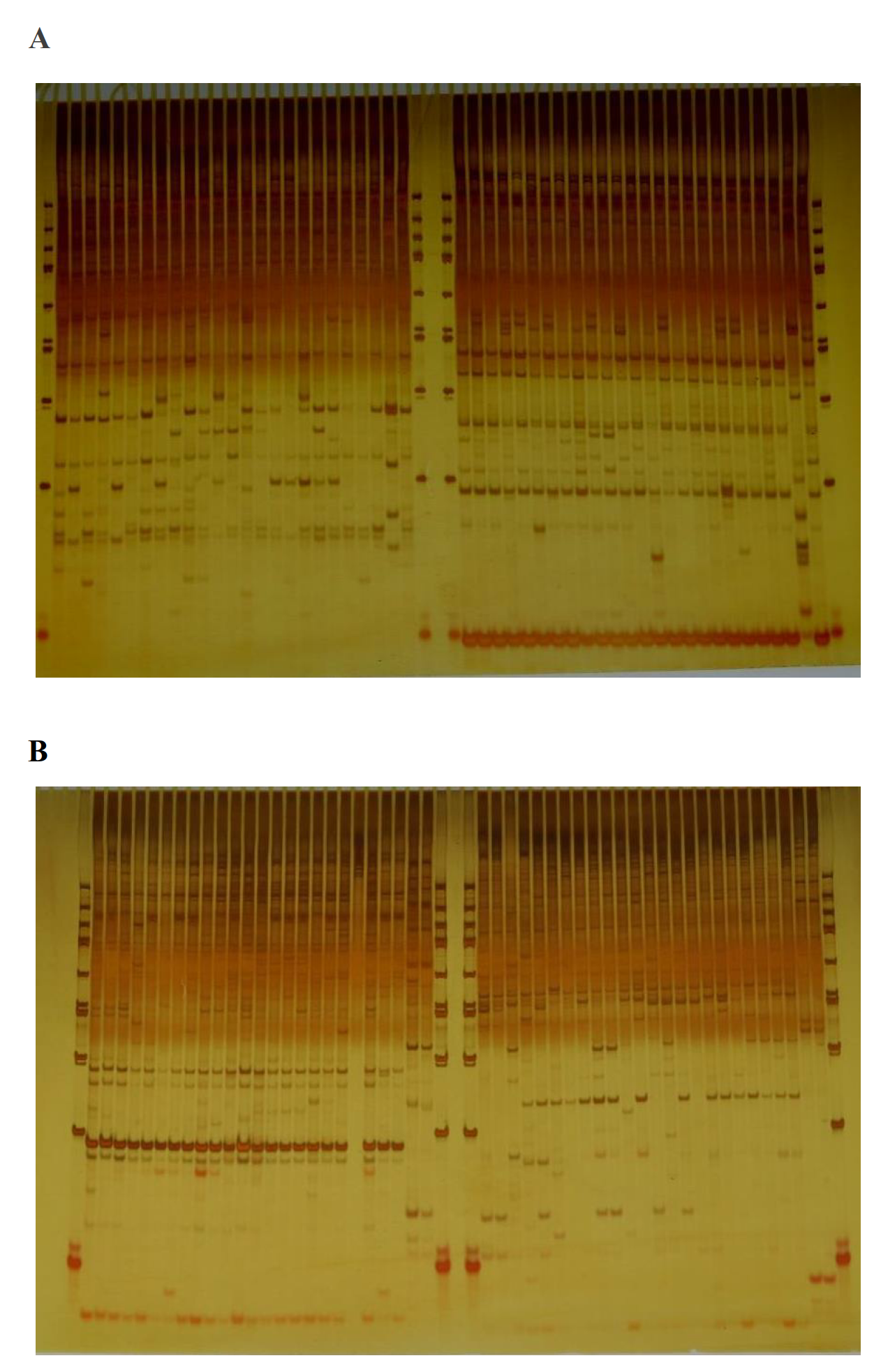

Supplement: Figure S2 — (A) M15 + E03 (left), M15 + E08 (right); (B) M9 + E14 (left), M9 + E20 (right) (accessions 1 to 23 from left to right). [file peerj-11-15900-s002.png]

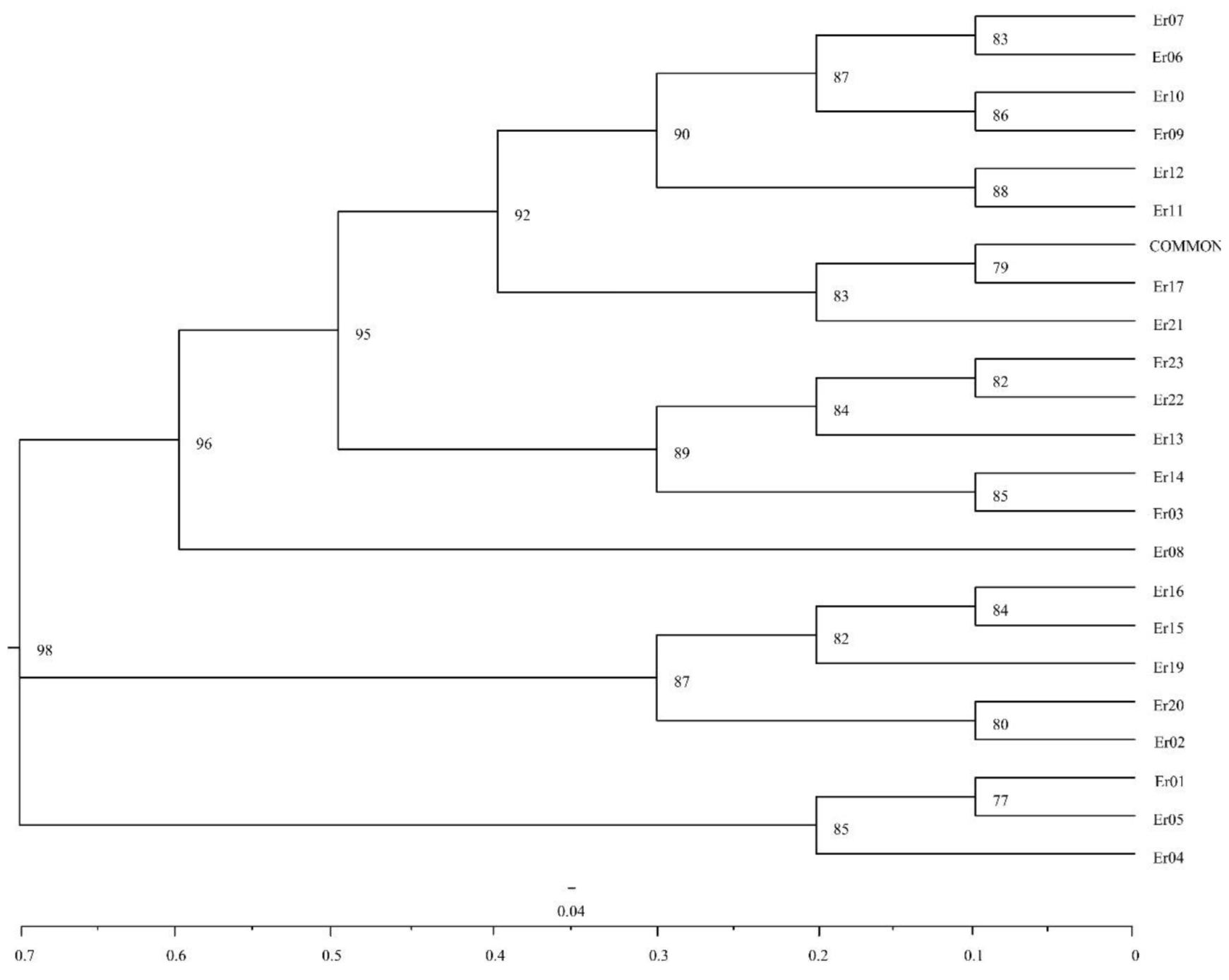

Supplement: Figure S3 [file peerj-11-15900-s003.png]

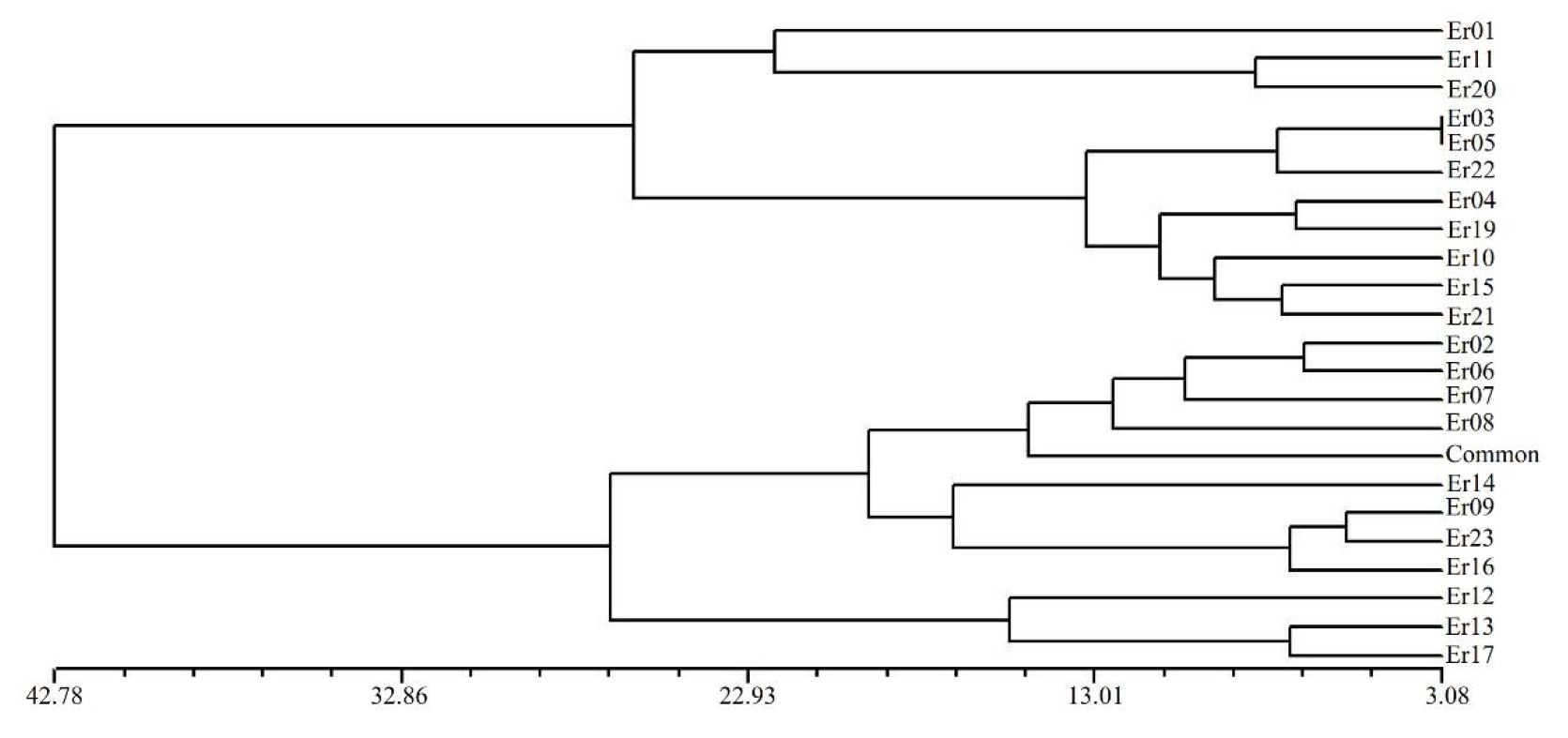

Supplement: Figure S4 [file peerj-11-15900-s004.png]

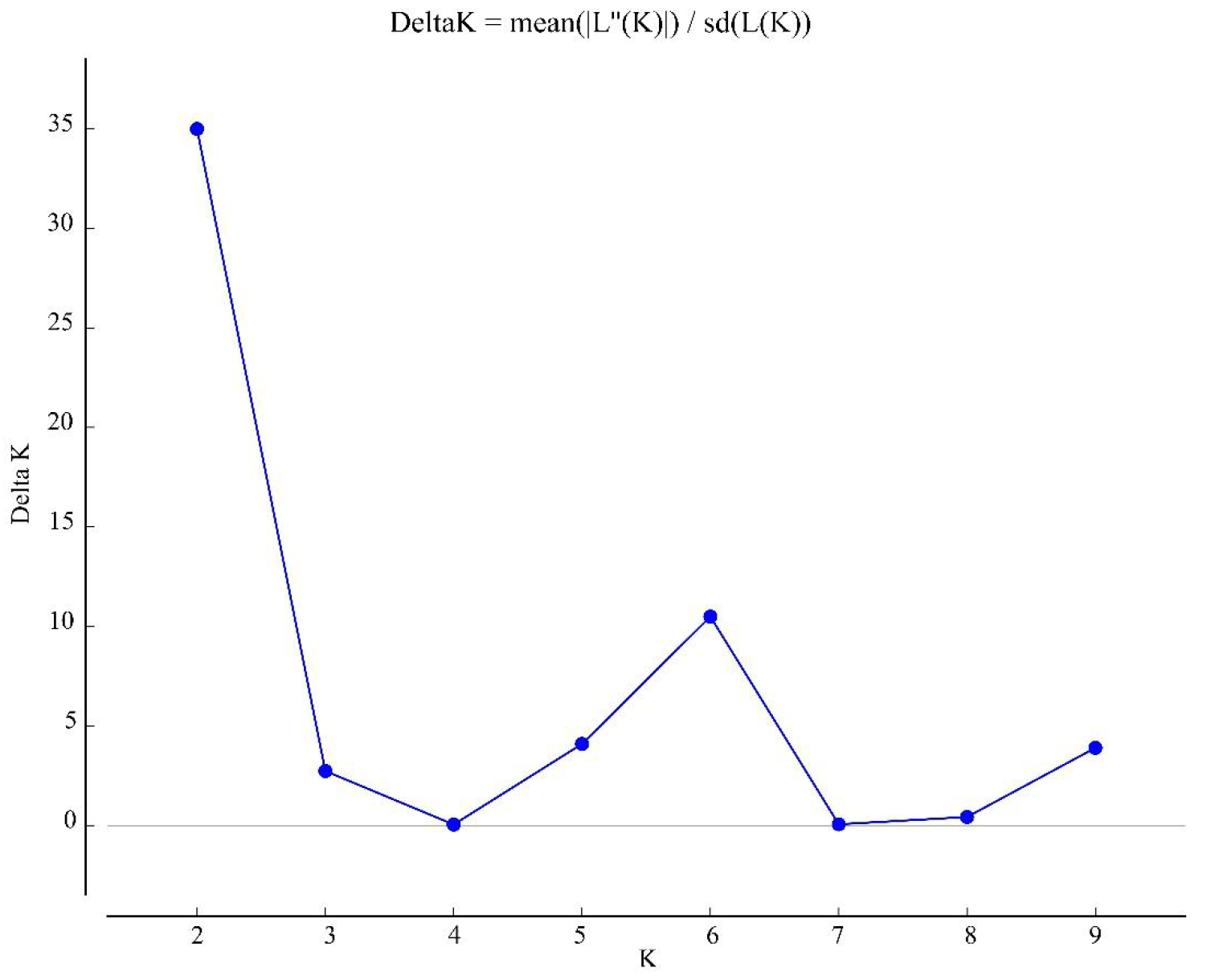

Supplement: Figure S5 [file peerj-11-15900-s005.png]
