# Supplementary material for: Genetic diversity analysis and molecular characteristics of wild centipedegrass using sequence-related amplified polymorphism (SRAP) markers
Source: PeerJ. 2023 Aug 24;11:e15900. doi: 10.7717/peerj.15900 (PMC10460567; doi:10.7717/peerj.15900)
Supplement: Table S1 [file peerj-11-15900-s009.docx]

**Table S1**. Accession number and collection source of the centipedegrass.

| Number | Collection of place names | Longitude(°E) | Latitude(°N) | Altitute(m) | Average annual temperature(℃) | Annual precipitation(mm) |
| --- | --- | --- | --- | --- | --- | --- |
| Er01 | Chongqing Nanshan Park | 106°37'48'' | 106°37'48'' | 321 | 18.52 | 1093 |
| Er02 | Huilong, Qionglai, Sichuan | 103°22′12″ | 103°22′12″ | 546 | 16.98 | 1447 |
| Er03 | Xinjin, Sichuan, | 102°54′00″ | 102°54′00″ | 2637 | 7.87 | 1065 |
| Er04 | Leshan, Sichuan | 103°45'36'' | 103°45'36'' | 375 | 17.53 | 1366 |
| Er05 | Longchang, Sichuan | 105°15'00'' | 105°15'00'' | 388 | 17.87 | 1073 |
| Er06 | Fuzhou Forest Park | 116°12'00'' | 116°12'00'' | 226 | 11.23 | 649 |
| Er07 | Hangzhou Zhejiang | 120°56'24'' | 120°56'24'' | 3 | 15.52 | 1060 |
| Er08 | Longxi, Chongqing | 106°45'00'' | 106°45'00'' | 406 | 17.89 | 1111 |
| Er09 | Conghua, Guangdong | 113°37'12'' | 113°37'12'' | 265 | 20.57 | 2051 |
| Er10 | Yueyang, Hunan | 113°06'36'' | 113°06'36'' | 29 | 17.13 | 1303 |
| Er11 | Pengshui Chongqing | 108°15'36'' | 108°15'36'' | 852 | 14.6 | 1356 |
| Er12 | Hainan | 111°20'24'' | 111°20'24'' | 15.43 | 24.5 | 1639 |
| Er13 | shenzhen university | 114°07'48'' | 114°07'48'' | 33 | 22.75 | 2065 |
| Er14 | Yichang, Hubei | 111°33'36'' | 111°33'36'' | 180 | 16.51 | 1125 |
| Er15 | Meishan, Sichuan | 103°50'24'' | 103°50'24'' | 413 | 17.04 | 1303 |
| Er16 | Emei Huangwan Township | 103°27'00'' | 103°27'00'' | 553 | 17.35 | 1647 |
| Er17 | Yibin, Sichuan | 104°39'00'' | 104°39'00'' | 341 | 18.32 | 1157 |
| COMMON | Imported from abroad | 155°19'48'' | 155°19'48'' | 224 | 13.84 | 1176 |
| Er19 | Shifang, Sichuan | 104°01'12'' | 104°01'12'' | 916 | 14.95 | 984 |
| Er20 | Sichuan Mianzhu | 104°07'12'' | 104°07'12'' | 1557 | 12.05 | 977 |
| Er21 | Chongqing Xiushan (hillside) | 109°01'48'' | 109°01'48'' | 400 | 16.74 | 1268 |
| Er22 | Qianjiang, Chongqing | 108°42'36'' | 108°42'36'' | 688 | 15.34 | 1399 |
| Er23 | Chongqing Xiushan (River Beach) | 109°07'12'' | 109°07'12'' | 682 | 15.23 | 1327 |
