# Supplementary material for: Genetic diversity analysis and molecular characteristics of wild centipedegrass using sequence-related amplified polymorphism (SRAP) markers
Source: PeerJ. 2023 Aug 24;11:e15900. doi: 10.7717/peerj.15900 (PMC10460567; doi:10.7717/peerj.15900)
Supplement: Table S2 [file peerj-11-15900-s010.docx]

**Table S2**. Seven morphological traits measured in 23 centipedegrass.

| Accession | Stolon internode length(SIL) | Stolon internode diameter(SID) | Erect branch leaf length(EBLL) | Erect branch leaf width(EBLW) | Stolon leaf length(SLL) | Stolon leaf width(SLW) | Grass layer height(GLH) |
| --- | --- | --- | --- | --- | --- | --- | --- |
| Er01 | 27.06 | 2.03 | 94.22 | 4.73 | 28.48 | 4.42 | 48.27 |
| Er02 | 29.28 | 2.15 | 117.43 | 4.56 | 35.3 | 4.88 | 104.8 |
| Er03 | 21.85 | 1.81 | 81.19 | 4.62 | 29.06 | 3.96 | 70.12 |
| Er04 | 24.6 | 1.9 | 90.11 | 4.44 | 26.11 | 4.29 | 74.04 |
| Er05 | 22.84 | 1.92 | 79.88 | 4 | 27.35 | 4.37 | 71.94 |
| Er06 | 25.46 | 1.71 | 112.69 | 3.89 | 32.81 | 3.33 | 106.39 |
| Er07 | 20.79 | 1.95 | 111.12 | 4.54 | 36.34 | 4.26 | 99.78 |
| Er08 | 21.52 | 1.9 | 120.7 | 5.2 | 31.29 | 4.63 | 111.14 |
| Er09 | 18.65 | 1.79 | 101.63 | 4.69 | 30.61 | 5.13 | 95.03 |
| Er10 | 28.92 | 1.97 | 85 | 4.69 | 34.91 | 4.44 | 74.81 |
| Er11 | 24.94 | 1.83 | 109.31 | 4.81 | 29.32 | 4.43 | 60.29 |
| Er12 | 21.61 | 2.14 | 138.95 | 4.56 | 38.96 | 4.76 | 102.98 |
| Er13 | 23.29 | 2.04 | 123.42 | 5.29 | 44.03 | 4.59 | 98.94 |
| Er14 | 23.49 | 1.9 | 100.48 | 4.54 | 41.99 | 4.67 | 105.51 |
| Er15 | 20.4 | 2.15 | 86.19 | 4.9 | 38.39 | 4.53 | 78.87 |
| Er16 | 17.78 | 1.83 | 95.35 | 4.51 | 29.15 | 4.64 | 94.58 |
| Er17 | 19.51 | 1.95 | 126.67 | 4.84 | 44.99 | 4.65 | 104.32 |
| COMMON | 28.7 | 1.88 | 107.97 | 4.69 | 24 | 4.32 | 102.86 |
| Er19 | 26.92 | 1.84 | 95.06 | 4.58 | 30.66 | 4.52 | 75.31 |
| Er20 | 23.91 | 1.86 | 108.23 | 5.14 | 30.31 | 4.95 | 68.49 |
| Er21 | 21.12 | 2 | 88.66 | 4.36 | 32.52 | 4.8 | 74.8 |
| Er22 | 20.43 | 1.98 | 84.6 | 4.15 | 22.87 | 4.07 | 67.95 |
| Er23 | 23.02 | 1.76 | 100.99 | 4.42 | 27.82 | 4.14 | 97.29 |
| Mean+SE | 23.31±0.68 | 1.93±0.03 | 102.60±3.32 | 4.62±0.71 | 32.49±1.26 | 4.47±0.08 | 86.46±3.73 |
| SD | 3.27 | 0.12 | 15.92 | 0.34 | 6.03 | 0.38 | 17.9 |
| CV% | 14.05 | 6.28 | 15.52 | 7.39 | 18.57 | 8.42 | 20.71 |
| Range of Variation | 17.78-29.28 | 1.71-2.15 | 79.88-138.95 | 3.89-5.29 | 22.87-44.99 | 3.33-5.13 | 48.27-111.14 |
| Accession | Stolon internode length(SIL) | Stolon internode diameter(SID) | Erect branch leaf length(EBLL) | Erect branch leaf width(EBLW) | Stolon leaf length(SLL) | Stolon leaf width(SLW) | Grass layer height(GLH) |
| F value | 2.296 | 2.13 | 5.474 | 2.232 | 5.403 | 2.218 | 3.753 |
| Significance P | <0.01 | <0.01 | <0.01 | <0.01 | <0.01 | <0.01 | <0.01 |
