# Supplementary material for: Genetic diversity analysis and molecular characteristics of wild centipedegrass using sequence-related amplified polymorphism (SRAP) markers
Source: PeerJ. 2023 Aug 24;11:e15900. doi: 10.7717/peerj.15900 (PMC10460567; doi:10.7717/peerj.15900)
Supplement: Table S3 [file peerj-11-15900-s011.docx]

**Table S3.** Primer name and sequence of SRAP.

| Primer name | Sequence（5′→3′） | Primer name | Sequence（5′→3′） |
| --- | --- | --- | --- |
| Me01 | TAGGTCCAAACCGGAAG | Em01 | GACTGCGTACGAATTAAG |
| Me06 | TAGGTCCAAACCGGCAC | Em02 | GACTGCGTACGAATTACA |
| Me07 | TAGGTCCAAACCGGCCA | Em03 | GACTGCGTACGAATTACT |
| Me09 | TAGGTCCAAACCGGCGG | Em04 | GACTGCGTACGAATTAGA |
| Me10 | TAGGTCCAAACCGGCTC | Em05 | GACTGCGTACGAATTATC |
| Me11 | TAGGTCCAAACCGGCTT | Em06 | GACTGCGTACGAATTCAC |
| Me12 | TAGGTCCAAACCGGGAT | Em07 | GACTGCGTACGAATTCCA |
| Me14 | TAGGTCCAAACCGGGGT | Em08 | GACTGCGTACGAATTCCG |
| Me15 | TAGGTCCAAACCGGGTA | Em09 | GACTGCGTACGAATTCGG |
| Me16 | TAGGTCCAAACCGGTAG | Em10 | GACTGCGTACGAATTCTC |
| Me17 | TAGGTCCAAACCGGTAT | Em12 | GACTGCGTACGAATTGAT |
| Me18 | TAGGTCCAAACCGGTCG | Em14 | GACTGCGTACGAATTGGT |
| Me19 | TAGGTCCAAACCGGTCT | Em15 | GACTGCGTACGAATTGTA |
| Me20 | TAGGTCCAAACCGGTTC | Em19 | GACTGCGTACGAATTTCT |
|  |  | Em20 | GACTGCGTACGAATTTTC |
