# Supplementary material for: Genetic diversity analysis and molecular characteristics of wild centipedegrass using sequence-related amplified polymorphism (SRAP) markers
Source: PeerJ. 2023 Aug 24;11:e15900. doi: 10.7717/peerj.15900 (PMC10460567; doi:10.7717/peerj.15900)
Supplement: Table S4 [file peerj-11-15900-s012.docx]

**Table S4.** Eigen Values by Axis and Sample Eigen Vectors.

| Axis No. | 1 | 2 | 3 | 4 | 5 | 6 | 7 | 8 | 9 | 10 |
| --- | --- | --- | --- | --- | --- | --- | --- | --- | --- | --- |
| EigenValue | 0.106 | 0.097 | 0.095 | 0.081 | 0.079 | 0.077 | 0.074 | 0.072 | 0.069 | 0.063 |
| Er01 | 0.103 | -0.058 | 0.014 | 0.011 | 0.076 | -0.034 | -0.046 | 0.041 | 0.017 | -0.017 |
| Er02 | 0.138 | -0.040 | 0.022 | -0.017 | 0.025 | 0.006 | -0.055 | 0.054 | 0.011 | -0.032 |
| Er03 | 0.016 | -0.079 | 0.081 | -0.020 | 0.076 | -0.011 | -0.025 | 0.053 | 0.017 | -0.002 |
| Er04 | 0.106 | -0.039 | -0.029 | 0.085 | -0.094 | 0.132 | 0.005 | 0.005 | -0.026 | 0.082 |
| Er05 | 0.110 | -0.008 | 0.027 | -0.022 | -0.081 | 0.025 | 0.023 | 0.005 | 0.029 | -0.035 |
| Er06 | -0.052 | -0.057 | -0.030 | 0.045 | -0.169 | -0.082 | -0.041 | -0.038 | 0.047 | 0.015 |
| Er07 | -0.036 | -0.074 | -0.033 | -0.027 | -0.047 | -0.143 | -0.010 | 0.029 | -0.111 | 0.010 |
| Er08 | 0.014 | -0.067 | 0.017 | -0.071 | -0.002 | -0.017 | -0.067 | -0.063 | -0.017 | 0.009 |
| Er09 | -0.014 | -0.081 | 0.021 | -0.121 | 0.016 | -0.012 | 0.133 | -0.036 | 0.066 | -0.002 |
| Er10 | -0.057 | -0.074 | 0.024 | 0.041 | 0.008 | 0.049 | 0.110 | -0.090 | 0.003 | -0.070 |
| Er11 | -0.080 | -0.037 | 0.028 | 0.128 | 0.045 | 0.045 | -0.033 | -0.002 | -0.079 | -0.075 |
| Er12 | -0.033 | -0.052 | -0.050 | 0.054 | 0.108 | 0.010 | 0.010 | -0.073 | -0.030 | 0.114 |
| Er13 | -0.033 | 0.025 | -0.062 | 0.054 | 0.042 | -0.028 | -0.077 | -0.008 | 0.112 | 0.012 |
| Er14 | 0.000 | 0.030 | -0.106 | 0.040 | 0.016 | -0.037 | 0.132 | 0.150 | -0.005 | 0.047 |
| Er15 | 0.045 | 0.082 | -0.095 | 0.027 | 0.022 | -0.028 | 0.006 | -0.084 | 0.049 | -0.058 |
| Er16 | 0.024 | 0.096 | -0.090 | -0.011 | 0.021 | -0.019 | -0.005 | -0.017 | 0.037 | -0.052 |
| Er17 | -0.096 | 0.001 | -0.075 | -0.067 | -0.030 | 0.099 | -0.029 | 0.081 | -0.032 | -0.092 |
| COMMON | -0.081 | 0.028 | -0.062 | -0.124 | 0.003 | 0.076 | -0.071 | -0.004 | -0.021 | 0.061 |
| Er19 | 0.060 | 0.110 | 0.014 | -0.051 | 0.025 | -0.001 | 0.017 | -0.059 | -0.102 | 0.030 |
| Er20 | 0.040 | 0.119 | 0.078 | 0.008 | -0.032 | -0.041 | 0.023 | -0.029 | -0.071 | -0.003 |
| Er21 | -0.048 | 0.055 | 0.093 | 0.028 | -0.001 | -0.020 | 0.004 | 0.032 | -0.012 | -0.050 |
| Er22 | -0.031 | 0.049 | 0.104 | -0.008 | -0.031 | 0.058 | 0.009 | 0.017 | 0.063 | 0.044 |
| Er23 | -0.097 | 0.073 | 0.109 | 0.019 | 0.004 | -0.027 | -0.014 | 0.038 | 0.056 | 0.064 |
