# Supplementary material for: Genetic diversity analysis and molecular characteristics of wild centipedegrass using sequence-related amplified polymorphism (SRAP) markers
Source: PeerJ. 2023 Aug 24;11:e15900. doi: 10.7717/peerj.15900 (PMC10460567; doi:10.7717/peerj.15900)
Supplement: Table S5 [file peerj-11-15900-s013.docx]

**Table S4.** Q value of 23 cetipedegrass accessions in two groups.

| Code | Q1 | Q2 | Group | Code | Q1 | Q2 | Group |
| --- | --- | --- | --- | --- | --- | --- | --- |
| Er01 | 0.898 | 0.102 | 2 | Er13 | 0.0498 | 0.9502 | 1 |
| Er02 | 0.8994 | 0.1006 | 2 | Er14 | 0.3218 | 0.6782 | 1 |
| Er03 | 0.3176 | 0.6824 | 1 | Er15 | 0.6082 | 0.3918 | 2 |
| Er04 | 0.8986 | 0.1014 | 2 | Er16 | 0.6053 | 0.3947 | 2 |
| Er05 | 0.9071 | 0.0929 | 2 | Er17 | 0.0038 | 0.9962 | 1 |
| Er06 | 0.1369 | 0.8631 | 1 | COMMON | 0.005 | 0.995 | 1 |
| Er07 | 0.1781 | 0.8219 | 1 | Er19 | 0.653 | 0.347 | 2 |
| Er08 | 0.2269 | 0.7731 | 1 | Er20 | 0.6847 | 0.3153 | 2 |
| Er09 | 0.1424 | 0.8576 | 1 | Er21 | 0.107 | 0.893 | 1 |
| Er10 | 0.1618 | 0.8382 | 1 | Er22 | 0.1294 | 0.8706 | 1 |
| Er11 | 0.1118 | 0.8882 | 1 | Er23 | 0.1015 | 0.8985 | 1 |
| Er12 | 0.111 | 0.889 | 1 |  |  |  |  |
