# Supplementary material for: Genetic diversity analysis and molecular characteristics of wild centipedegrass using sequence-related amplified polymorphism (SRAP) markers
Source: PeerJ. 2023 Aug 24;11:e15900. doi: 10.7717/peerj.15900 (PMC10460567; doi:10.7717/peerj.15900)
Supplement: Table S6 [file peerj-11-15900-s014.docx]

**Table S5.** Distribution of the Q value of cetipedegrass germplasm in the 3 geographical groups.

|  | NO. | Q1>0.8 | Q2>0.8 | Q<0.8 |
| --- | --- | --- | --- | --- |
| SC | 9 | 3 | 2 | 44.40% |
| CQ | 6 | 1 | 4 | 16.70% |
| OT | 8 | 0 | 6 | 25.00% |
| Total | 23 | 4 | 12 | 30.40% |

SC: from Sichuan province, CQ: from Chongqing municipality, OT: Other accessions except Sichuan and Chongqing.
