# Supplementary material for: Genetic diversity analysis and molecular characteristics of wild centipedegrass using sequence-related amplified polymorphism (SRAP) markers
Source: PeerJ. 2023 Aug 24;11:e15900. doi: 10.7717/peerj.15900 (PMC10460567; doi:10.7717/peerj.15900)
Supplement: Table S7 — SC: from Sichuan province, CQ: from Chongqing municipality, OT: Other accessions except Sichuan and Chongqing. [file peerj-11-15900-s015.docx]

**Table S7.** Correlation data of seven morphological traits and geographical groups Mental analysis.

|  | SC | | CQ | | OT | | Total | |
| --- | --- | --- | --- | --- | --- | --- | --- | --- |
|  | r | p | r | p | r | p | r | p |
| SIL | 0.2465 | 0.1246 | -0.0533 | 0.4916 | 0.0211 | 0.4435 | 0.0857 | 0.2055 |
| SID | 0.0441 | 0.4477 | 0.1284 | 0.2777 | -0.3198 | 0.1057 | -0.0527 | 0.3265 |
| EBLL | 0.2761 | 0.0754 | -0.2251 | 0.2375 | 0.3492 | 0.1583 | 0.0074 | 0.4552 |
| EBLW | -0.0439 | 0.4313 | 0.2081 | 0.2708 | -0.3605 | 0.1095 | -0.1160 | 0.1851 |
| SLL | 0.3177 | 0.0807 | 0.2111 | 0.2888 | -0.0753 | 0.4244 | 0.1925 | 0.0595 |
| SLW | -0.3228 | 0.0754 | 0.4370 | 0.0916 | -0.6496 | 0.1206 | -0.2108 | 0.0529 |
| GLH | 0.3803 | 0.0524 | -0.2159 | 0.2361 | 0.6509 | 0.0737 | 0.0276 | 0.3563 |

SC: from Sichuan province, CQ: from Chongqing municipality, OT: Other accessions except Sichuan and Chongqing.
