# Supplementary material for: Genetic diversity analysis and molecular characteristics of wild centipedegrass using sequence-related amplified polymorphism (SRAP) markers
Source: PeerJ. 2023 Aug 24;11:e15900. doi: 10.7717/peerj.15900 (PMC10460567; doi:10.7717/peerj.15900)
Supplement: Table S8 [file peerj-11-15900-s016.docx]

**Table S6.** Correlation data of climate data and geographical groups Mental analysis.

|  | SC | | CQ | | OT | | Total | |
| --- | --- | --- | --- | --- | --- | --- | --- | --- |
|  | r | p | r | p | r | p | r | p |
| BIO1 | 0.2298 | 0.0771 | 0.2677 | 0.1833 | -0.3235 | 0.0881 | 0.1267 | 0.1627 |
| BIO2 | 0.0845 | 0.3201 | 0.3895 | 0.0028 | 0.1303 | 0.2637 | 0.1831 | 0.1127 |
| BIO3 | -0.0092 | 0.4095 | 0.4859 | 0.0361 | 0.0098 | 0.4530 | 0.1511 | 0.1299 |
| BIO4 | 0.5064 | 0.0037 | 0.1354 | 0.2361 | 0.0544 | 0.4017 | 0.1590 | 0.1060 |
| BIO5 | 0.1685 | 0.2298 | 0.2096 | 0.1889 | 0.1261 | 0.2403 | 0.1007 | 0.2285 |
| BIO6 | 0.2652 | 0.0420 | 0.1225 | 0.3069 | -0.1665 | 0.2333 | 0.1206 | 0.1628 |
| BIO7 | 0.4690 | 0.0049 | -0.2071 | 0.0042 | -0.0716 | 0.3650 | 0.1109 | 0.1909 |
| BIO8 | 0.2471 | 0.0645 | 0.2839 | 0.0806 | 0.0313 | 0.4492 | 0.1582 | 0.1102 |
| BIO9 | 0.2936 | 0.0301 | 0.1111 | 0.3319 | -0.2882 | 0.0979 | 0.1449 | 0.1106 |
| BIO10 | 0.1754 | 0.2085 | 0.1701 | 0.2278 | 0.2412 | 0.1058 | 0.0966 | 0.2330 |
| BIO11 | 0.2936 | 0.0310 | 0.2306 | 0.2472 | -0.2447 | 0.1504 | 0.1536 | 0.1084 |
| BIO12 | -0.3460 | 0.0344 | 0.2825 | 0.0472 | -0.3725 | 0.0347 | 0.1894 | 0.0777 |
| BIO13 | -0.0745 | 0.3245 | 0.1117 | 0.2597 | -0.0523 | 0.3809 | 0.1415 | 0.1107 |
| BIO14 | 0.4445 | 0.0033 | 0.2880 | 0.1569 | 0.1808 | 0.1918 | 0.2513 | 0.0066 |
| BIO15 | 0.3322 | 0.0330 | 0.4333 | 0.0083 | 0.3290 | 0.0536 | 0.2623 | 0.0434 |
| BIO16 | -0.1821 | 0.1980 | 0.1364 | 0.2403 | -0.0682 | 0.3580 | 0.1586 | 0.0975 |
| BIO17 | 0.4686 | 0.0010 | 0.2660 | 0.1681 | 0.1841 | 0.1836 | 0.2354 | 0.0141 |
| BIO18 | -0.1441 | 0.2356 | 0.1881 | 0.1722 | -0.0743 | 0.3629 | 0.2013 | 0.0586 |
| BIO19 | 0.4686 | 0.0009 | 0.1908 | 0.1889 | -0.1116 | 0.2836 | 0.1641 | 0.0788 |

SC: from Sichuan province, CQ: from Chongqing municipality, OT: Other accessions except Sichuan and Chongqing.
